# Chemical synthesis and enzymatic, stereoselective hydrolysis of a functionalized dihydropyrimidine for the synthesis of *β*-amino acids

**DOI:** 10.1186/s13568-015-0174-8

**Published:** 2015-12-24

**Authors:** Christin Slomka, Sabilla Zhong, Anna Fellinger, Ulrike Engel, Christoph Syldatk, Stefan Bräse, Jens Rudat

**Affiliations:** Section II: Technical Biology, Institute of Process Engineering in Life Sciences, Karlsruhe Institute of Technology (KIT), Engler-Bunte-Ring 1, 76131 Karlsruhe, Germany; Institute of Organic Chemistry, Karlsruhe Institute of Technology (KIT), Fritz-Haber-Weg 6, 76131 Karlsruhe, Germany; Institute of Toxicology and Genetics, Institute of Technology (KIT), Hermann-von Helmholtz-Platz 1, 76344, Eggenstein-Leopoldshafen, Germany

**Keywords:** β-Amino acids, Dihydrouracil, Enantioselectivity, Enzyme catalysis, Hydrolases

## Abstract

A novel substrate, 6-(4-nitrophenyl)dihydropyrimidine-2,4(1*H*,3*H*)-dione (*p*NO_2_PheDU), was chemically synthesized and analytically verified for the potential biocatalytic synthesis of enantiopure *β*-amino acids. The hydantoinase (EC 3.5.2.2) from *Arthrobacter crystallopoietes* DSM20117 was chosen to prove the enzymatic hydrolysis of this substrate, since previous investigations showed activities of this enzyme toward 6-monosubstituted dihydrouracils. Whole cell biotransformations with recombinant *Escherichia coli* expressing the hydantoinase showed degradation of *p*NO_2_PheDU. Additionally, the corresponding *N*-carbamoyl-*β*-amino acid (*N*Carb*p*NO_2_*β*Phe) was chemically synthesized, an HPLC-method with chiral stationary phases for detection of this product was established and thus *(S)*-enantioselectivity toward *p*NO_2_PheDU has been shown. Consequently this novel substrate is a potential precursor for the enantiopure *β*-amino acid *para*-nitro-*β*-phenylalanine (*p*NO_2_*β*Phe).

**Electronic supplementary material:**

The online version of this article (doi:10.1186/s13568-015-0174-8) contains supplementary material, which is available to authorized users.

## Introduction

The use of enantiopure *β*-amino acids is of increasing importance, since they are constituents of several biologically active secondary metabolites such as taxol, jaspamide, theopalauamide and dolastatins (Weiner et al. 2010). As building blocks for *β*-peptides, which are able to form very stable and predictable secondary structures, they are also promising in applications as peptidomimetics (Cheng et al. 2001; Frackenpohl et al. 2001; Seebach and Gardiner 2008). Furthermore, cyclized *β*-amino acids like *β*-lactams show encouraging pharmacological properties (Magriotis 2001), but also some *β*-amino acids in their free form (Juaristi 2005).

Chemical methods for the synthesis of *β*-amino acids attracted attention over the last decade, essentially based on classical resolution, stoichiometric use of chiral auxiliaries and homologation of *α*-amino acids (Liu and Sibi 2002; Juaristi 2005). However, when applying in an industrial scale, these strategies show limitations as resolutions of racemic mixtures are time consuming and cause high costs (Liu and Sibi 2002; Weiner et al. 2010). Alternatively, promising biocatalytical routes for the synthesis of enantiopure *β*-amino acids, as the application of transaminases (Rudat et al. 2012), monooxygenases (Rehdorf et al. 2010) or aminomutases (Wu et al. 2009) have been investigated. Most frequently, amino and ester functionalities in substrates are utilized for kinetic resolutions with hydrolytic enzymes. To date, the best exploited enzymes for the synthesis of *β*-amino acids are lipases, for example cleaving *N*-acetylated *β*-amino acids or racemic *β*-amino acid esters (Liljeblad and Kanerva 2006; Tasnádi et al. 2008).

**Fig. 1:**
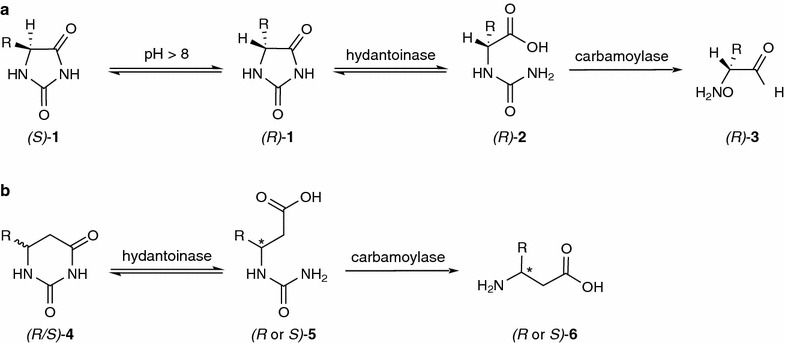
**a** Hydantoinase process for the synthesis of *(R)*-*α*-amino acids starting from racemic 5-monosubstituted hydantoins. **b** Proposed modified hydantoinase process for the synthesis of enantiopure *β*-amino acids starting from racemic 6-monosubstituted dihydrouracils

Since kinetic resolutions merely enable a maximum yield of 50 %, lately the application of a modified hydantoinase process was proposed (Fig. 1b) (Engel et al. 2014). The latter is based on the classical hydantoinase process (Fig. 1a), which is well established in industry for the production of enantiopure *α*-amino acids **3** as *α*-*(R)*-phenylglycine and *α*-*(R)*-*p*-hydroxyphenylglycine as side chains of the semisynthetic antibiotics ampicillin and amoxicillin (May et al. 2000; Bommarius et al. 2001). To date, whole cell biocatalysis is widely used due to low production costs and simple separation of catalyst and product (Slomka et al. 2014). Together with the application of hydantoin racemases or spontaneous racemization of unreacted substrates (hydantoins, **1**) under slightly alkaline conditions (Ware 1950; Kato et al. 1987; Las Heras-Vazquez et al. 2009), the enantioselectivity of the involved hydantoinase as well as carbamoylase cleaving the *N*-carbamoyl-*α*-amino acid **2** leads to a dynamic kinetic resolution and therefore a maximum yield of 100 %.

Compared to the hydrolysis of hydantoins toward *α*-amino acids, the hydrolysis of dihydropyrimidines **4** to their *N*-carbamoyl derivatives **5** as a route to *β*-amino acids **6** has not been extensively studied yet. In 1998, May et al. showed the hydrolysis of dihydrouracil by a hydantoinase of *Arthrobacter aurescens* and later this hydantoinase was successfully employed to hydrolyse 6-phenyldihydrouracil (May et al. 1998; Servi et al. 2005). Another group investigated the activity of the former commercially available hydantoinase from *Vigna vulgaris* toward differently substituted dihydrouracils (O’Neill et al. 2011). Even the second step of the modified hydantoinase process was realized by Martínez-Goméz et al.: The synthesis of *α*-methyl-*β*-alanine from 5-methyl-5,6-dihydrouracil was accomplished using the dihydropyrimidinase from *Sinorhizobium meliloti* CECT4114 and the *β*-ureidopropionase from *Agrobacterium tumefaciens* C58 (Martínez-Gómez et al. 2012).

In contrast to the classical hydantoinase process, one of the main challenging parameters of the process development using 6-monosubstituted 5,6-dihydropyrimidines as substrates is to achieve a yield of 100 % of enantiopure product, since their chemical racemization is not yet known to occur and no suitable racemase is known (Martínez-Gómez et al. 2012). Given that the viability of this route to *β*-amino acids was proven in some prior investigations (Engel et al. 2012a, b; O’Neill et al. 2011), in this work we chemically synthesized a novel substrate, 6-(4-nitrophenyl)dihydropyrimidine-2,4(1*H*,3*H*)-dione (*p*NO_2_PheDU (*R/S)*-**4a**) and proved the hydrolysis of this compound by the hydantoinase from the gram positive soil bacterium *A. crystallopoietes* DSM20117. The enzyme was found to be enantioselective for this reaction, making *p*NO_2_PheDU (*R/S*)-**4a**) a promising precursor, since the resulting *β*-amino acid may offer specific properties itself as for example known for the β-amino α-hydroxy acid which is part of the antitumor agent paclitaxel (Taxol™) or regarding downstream chemistry (Fleming et al. 1993).

## Materials and methods

Chemicals were of reagent grade and obtained from commercial sources if not stated otherwise. *(R)*-3-Amino-3-(4-nitrophenyl)-propionic acid (*(R)*-*para*-nitro-*β*-phenylalanine) was purchased from Pep-Tech Corporation (Burlington, USA).

### Media

The medium for cultivation and induction was lysogeny broth (LB) medium containing 10 g/L tryptone, 5 g/L yeast extract and 10 g/L NaCl. The pH was adjusted to 7 with NaOH.

### Bacterial strain, plasmid and expression

*Escherichia coli* JM109 harboring the plasmid pJAVI2 (Werner et al. 2004) was cultured overnight in 20 mL LB medium containing ampicillin (100 µg/mL) in a shaking incubator at 37 °C and 120 rpm. The resulting preculture was added to 200 mL LB-medium in a 1 L shaking flask to an OD_600_ of 0.1 and incubated at 37 °C and 120 rpm. Induction was carried out at an OD_600_ of 0.4–0.6 by adding rhamnose (2 g/L final concentration) and subsequently the cultivation was carried out at 30 °C and 120 rpm for an additional 6 h. After induction time, the cells were harvested by centrifugation (4700×*g*, 15 min, 4 °C), treated with liquid nitrogen and stored at −20 °C.

### Assay of enzyme activity

For whole cell biotransformation reactions, the harvested *E. coli* cells were thawed on ice, washed twice with Tris–HCl buffer (50 mM, pH 8) and resuspended in the same buffer (ratio of 10 mL buffer for 100 mL of harvested *E. coli* culture). Due to the poor water solubility, the substrate solution was prepared by dissolving *p*NO_2_PheDU (40 mM) in DMSO and afterwards diluting to a concentration of 4 mM with Tris–HCl buffer (50 mM, pH 8). The product solution of *N*Carb*p*NO_2_βPhe for analytical purposes was prepared in the same way. For starting the biotransformation reaction, 750 µL of the cell suspension was added to 750 µL of the prewarmed substrate solution to obtain a starting concentration of 2 mM *p*NO_2_PheDU (resulting in 25 mg cells per 1.5 mL reaction mixture). The assay was carried out in a thermomixer (Eppendorf) at 40 °C and 800 rpm for 24 h. Samples were taken at selected reaction times by withdrawing 200 µL from the reaction mixture, centrifugation (13,000 rpm, 5 min) and storage of the supernatant at −20 °C until analysis. For the determination of cell dry weight, 1.5 mL Eppendorf cups were dried overnight at 60 °C and subsequently weighed (triplicate). Thereafter, 1 mL of cell suspension was centrifuged (13,000 rpm, 5 min) in these reaction vessels and again dried overnight at 60 °C. After discarding of the supernatant, the cell dry weight was determined.

### Analytical procedures

^*1*^*H* *NMR* spectra were recorded on a BRUKER Avance 300 (300 MHz) or a BRUKER Avance 400 (400 MHz) device as solutions at room temperature. Chemical shifts are expressed in parts per million (ppm, δ), downfield from tetramethylsilane (TMS) and referenced to residual DMSO-d_5_ (2.50 ppm) as internal standard. All coupling constants are absolute values and *J* values are expressed in Hertz (Hz). The spectra were analyzed according to first order and the descriptions of signals include: s = singlet, d = doublet, dd = doublet of doublets, t = triplet, q = quartet, m = multiplet.

^*13*^*C* *NMR* spectra were recorded on a BRUKER Avance 300 (75 MHz) or a BRUKER Avance 400 (100 MHz) device as solutions at room temperature. Chemical shifts are expressed in parts per million (ppm, δ), downfield from tetramethylsilane (TMS) and referenced to DMSO-d_6_ (39.5 ppm) as internal standard. The signal structure was analyzed by DEPT and is described as follows: + = primary or tertiary C-atom (positive signal), − = secondary C-atom (negative signal), and C_q_ = quaternary C-atom (no signal).

*Electron ionization mass spectrometry* (*EI–MS*) and fast atom bombardment mass spectrometry (FAB-MS) was performed by using a *Finnigan* MAT 90 (70 eV). The molecular fragments are quoted as the relation between mass and charge (*m/z*), the intensities as a percentaged value relative to the intensity of the base signal (100 %). The abbreviation [M]^+^ refers to the molecule ion and [M + H]^+^ refers to the protonated molecule ion.

*Infrared spectroscopy* (IR) data were recorded on FT–IR *Bruker* IFS 88 and are reported as follows: frequency of absorption (cm^−1^), intensity of absorption (vs = very strong, s = strong, m = medium, w = weak, vw = very weak, br = broad).

*Elemental analysis* (EA) was carried out using an ELEMENTAR vario MICRO device. The values for carbon (C), hydrogen (H), and nitrogen (N) are expressed in weight percent.

*HPLC analysisp*NO_2_PheDU and *N*Carb*p*NO_2_βPhe were analyzed by HPLC on an Agilent 1200 system (Agilent Technology, Santa Clara, USA) using a HyperClone ODS-C18 column (5 µm, 120 Å, 50 × 4.6 mm, Phenomenex). 5 µL of the sample were injected without any dilution. A gradient flow method with a 0.8 mL/min flow rate was used. The initial mobile phase was composed of 5 % (v/v) acetonitrile acidified with 0.5 % (v/v) trifluoroacetic acid and 95 % (v/v) bidest. water. From 0 to 25 min, the acetonitrile ratio was increased to 10 %, afterwards from 25 to 26 min it was lowered to 5 % again. The detection wavelength was 257 nm and the column temperature 22 °C.

Chiral analysis of *N*Carb*p*NO_2_βPhe was carried out utilizing a Chiralpak QN-AX column (5 µm, 150 × 46 mm, Daicel, Chiral Technologies Europe, France). An isocratic flow method with 0.3 mL/min was used; the mobile phase consisted of 98 % (v/v) methanol (0.2 % v/v ammonium formate) and 2 % (v/v) acetic acid (0.2 M, adjusted to pH 6 with ammonia). The detection wavelength was 257 nm, the column temperature was 30 °C and 5 µL of undiluted sample were injected.

### Preparation of *p*NO_2_PheDU and the corresponding *N*-carbamoyl derivative

#### 6-(4-nitrophenyl)dihydropyrimidine-2,4(1*H*,3*H*)-dione (*p*NO_2_PheDU, 4a) (Svĕtlík and Veizerová 2011)

4-Nitrobenzaldehyde (**7**, 613 mg, 5.00 mmol, 1.00 equiv.), urea (**8**, 300 mg, 5.00 mmol, 1.00 equiv.) and meldrum’s acid (**9**, 721 mg, 5.00 mmol, 1.00 equiv.) were suspended in acetic acid (10 mL) and refluxed for 6 h. The product precipitated overnight. The solvent was removed under reduced pressure and the residue was recrystallized from EtOH. *p*NO_2_PheDU was obtained as a yellowish powder (489 mg, 2.08 mmol, 42 %).

^**1**^**H** **NMR** (400 MHz, DMSO-d_6_): δ = 2.66 (dd, ^2^*J* = 16.4, ^3^*J* = 6.9 Hz, 1H, C*H*_2_), 2.91 (dd, ^2^*J* = 16.4, ^3^*J* = 5.9 Hz, 1H, C*H*_2_), 4.82–4.91 (m, 1H, C*H*), 7.62 (d, ^3^*J* = 8.8 Hz, 2H, 2 × C*H*_Ar_), 8.12 (s, 1H, N*H*), 8.25 (d, ^3^*J* = 8.8 Hz, 2H, 2 × C*H*_Ar_), 10.25 (s, 1H, N*H*) ppm. ^**13**^**C** **NMR** (100 MHz, DMSO-d_6_): δ = 37.7 (−, *C*H_2_), 49.7 (+, *C*H), 123.8 (+, *C*H_Ar_), 127.5 (+, 2 × *C*H_Ar_), 147.0 (C_q_, *C*NO_2_), 148.7 (C_q_, 2 × *C*_Ar_), 153.7 (C_q_, N(*C*O)N), 169.3 (C_q_, N(*C*O)C) ppm. **IR** (ATR): ν˜ = 3233 (vw), 3075 (w), 2846 (vw), 1702 (m), 1595 (vw), 1517 (w), 1487 (w), 1408 (w), 1342 (w), 1329 (w), 1283 (w), 1235 (w), 1212 (w), 1159 (w), 1108 (w), 1010 (vw), 991 (vw), 941 (vw), 849 (w), 764 (w), 731 (w), 691 (w), 644 (vw), 615 (w), 581 (w), 531 (w), 511 (w), 462 (vw), 441 (vw), 415 (w) cm^−1^. **MS** (EI, 70 eV), *m/z* (%): 236 (7) [M + H]^+^, 235 (48) [M]^+^, 218 (13) [M − OH]^+^, 177 (32), 164 (13), 151 (42), 149 (22), 119 (13), 113 (21), 107 (100) [C_6_H_6_NO]^+^, 103 (17) [C_4_H_5_N_2_O_2_]^+^, 91 (11) [C_7_H_7_]^+^, 77 (43), 70 (41), 60 (22). **HRMS** (EI, C_10_H_9_O_4_N_3_): calc. 235.0588; found 235.0589. **EA** (C_10_H_9_O_4_N_3_) calc. C 51.07 %, H 3.86 %, N 17.87 %; found C 50.87 %, H 3.72 %, N 17.62 %.

#### 3-(4-Nitrophenyl)-3-ureidopropanoic acid (*N*Carb*p*NO_2_βPhe, 5a)(Posner 1912)

4-Nitrophenylalanine (**10**, 488 mg, 2.38 mmol, 1.00 equiv.) was added to a solution of potassium cyanate (565 mg, 7.14 mmol, 3.00 equiv.) in H_2_O (8 mL). The mixture was heated to reflux for 1 h. After cooling to room temperature, the solution was acidified with diluted aqueous HCl solution. The *β*-carbamoyl amino acid *N*Carb*p*NO_2_βPhe precipitated as a yellowish solid (418 mg, 1.65 mmol, 71 %).

^**1**^**H** **NMR** (400 MHz, DMSO-d_6_): δ = 2.71 (d, ^3^*J* = 6.9 Hz, 2H, C*H*_2_), 5.08 (q, ^3^*J* = 7.2 Hz, 1H, C*H*), 5.64 (s, 2H, N*H*_2_), 6.74 (d, ^3^*J* = 8.4 Hz, 1H, N*H*), 7.58 (d, ^3^*J* = 8.6 Hz, 2H, 2 × C*H*_Ar_), 8.19 (d, ^3^*J* = 8.6 Hz, 2H, 2 × C*H*_Ar_) ppm. ^**13**^**C** **NMR** (100 MHz, DMSO-d_6_): δ = 40.9 (−, *C*H_2_), 49.9 (+, *C*H), 123.4 (+, *C*H_Ar_), 127.7 (+, 2 × *C*H_Ar_), 146.3 (C_q_, *C*_Ar_), 151.8 (C_q_, *C*_Ar_), 157.8 (C_q_, *C*O), 171.8 (C_q_, *C*O_2_H) ppm. **IR** (ATR): $\tilde{v}$ = 3390 (w), 1697 (m), 1631 (w), 1604 (w), 1506 (m), 1386 (w), 1345 (m), 1313 (m), 1210 (m), 1176 (w), 1105 (w), 1035 (w), 968 (w), 938 (w), 851 (m), 771 (vw), 751 (w), 700 (w), 649 (w), 625 (w), 555 (m), 472 (m), 448 (m) cm^−1^. **MS** (FAB, 3-NBA), *m/z* (%): 254 (23) [M + H]^+^, 233 (12), 192 (100). **HRMS** (FAB, (M^+^ + H), C_10_H_12_O_5_N_3_): calc. 254.0771; found 254.0772.

## Results

### Preparation of *p*NO_2_PheDU and the corresponding *N*-carbamoyl derivative

**Fig. 2:**
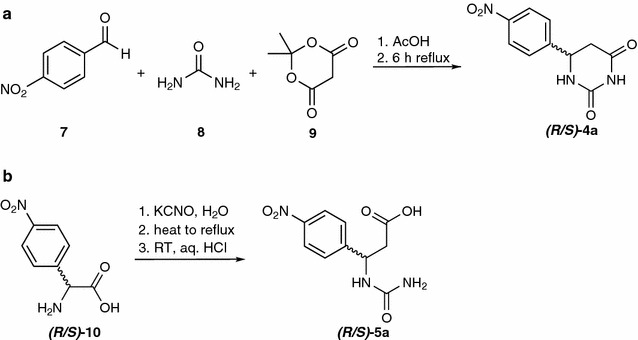
Synthesis of *p*NO_2_PheDU (**a**, *(R/S)*-4a) and the corresponding *N*-carbamoyl-*β*-amino acid (**b**, *(R/S)*-5a)

Several examinations already revealed the wide substrate scope of the hydantoinase of *A. crystallopoietes* DSM20117 concerning hydantoins (Siemann et al. 1999). Furthermore the hydrolysis of dihydrouracils was discovered, making this enzyme a potential tool toward enantiopure *β*-amino acids applying a modified hydantoinase process (Engel et al. 2012b). Synthesis of a novel non-natural dihydrouracil *p*NO_2_PheDU (*(R/S)*-**4a**) (Svĕtlík and Veizerová 2011) for hydrolysis by the hydantoinase of *A. crystallopoietes* as well as the corresponding product (*(R/S)*-**5a**) (Posner 1912) for analytical issues is shown in Fig. 2.

The synthesis of *p*NO_2_PheDU (*(R/S)*-**4a,** 489 mg, 2.08 mmol, 42 %) and the corresponding *N*-carbamoyl derivative *N*Carb*p*NO_2_βPhe (*(R/S)*-**5a,** 418 mg, 1.65 mmol, 71 %) was carried out and verified with the methods described above.

### Biocatalytic conversion of *p*NO_2_PheDU

After synthesis and analysis of the above mentioned compounds, whole cell biotransformation reactions with *E. coli* hosting the recombinantly expressed hydantoinase employing *p*NO_2_PheDU (*(R/S)*-**4a**) as substrate were performed. The reaction was carried out in Tris–HCl (50 mM, pH 8) at 40 °C and 800 rpm for 24 h. Table 1 shows the reaction course of *p*NO_2_PheDU concentration (*(R/S)*-**4a**) as well as conversion yields during the entire reaction time.

**Table 1:** Conversion of *p*NO_2_PheDU ((*R/S)*-4a) during 24 h whole cell biocatalysis with recombinant *E. coli* JM109 expressing the hydantoinase of *A. crystallopoietes*

Time (h)	*p*NO_2_PheDU^a^ (mM)	Conversion (%)
0	1.94 ± 0.03	–
1	1.63 ± 0.05	16
2	1.29 ± 0.04	34
3	1.24 ± 0.02	36
4	1.18 ± 0.04	39
5	1.16 ± 0.11	40
24	0.54 ± 0.05	72

^a^Initial substrate concentration: 2 mM. Reactions and measurements were carried out in triplicates. Detection by HPLC Agilent 1200 system (HyperClone ODS-C18 column; 257 nm, 22 °C, 0.8 mL/min; initial mobile phase: 5 % (v/v) acetonitrile acidified with 0.5 % (v/v) trifluoroacetic acid and 95 % (v/v) bidest. water, 0-25 min: acetonitrile ratio increased to 10 % (v/v), 25–26 min acetonitrile ratio lowered to 5 % (v/v)

The *p*NO_2_PheDU concentration (detected by HPLC) decreased from 1.94 to 1.63 mM within 1 h and finally to 0.54 mM after 24 h. Since control experiments performed without cells did not show a decrease of substrate concentration below the initial concentration of 2 mM, the mentioned results prove chemical stability as well as the enzymatic hydrolysis of *p*NO_2_PheDU by the investigated hydantoinase with a specific activity of 0.326 mU/mg_cdw_. The observed conversion yield for the hydrolysis of the novel substrate was 72 % after 24 h.

After successful hydrolysis of *p*NO_2_PheDU, we investigated the enantioselectivity of the applied hydantoinase regarding applications in pharmaceutical industries.

### Enantioselectivity of the hydantoinase from *A. crystallopoietes* for pNO_2_PheDU

**Fig. 3:**
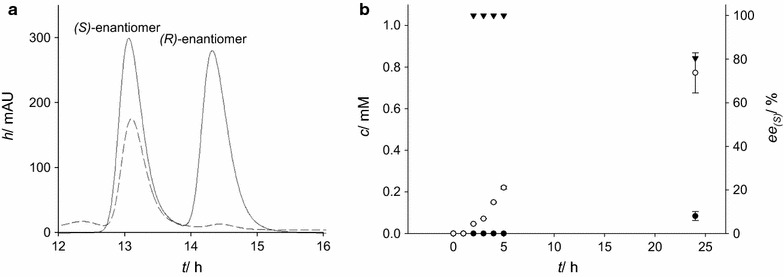
Studies on the enantioselectivity of the hydantoinase from *A. crystallopoietes* toward *p*NO_2_PheDU (*(R/S)*-4a). **a** Chiral separations in order to determine the enantioselectivity of the hydantoinase *p*NO_2_PheDU (*(R/S)*-4a) as substrate in whole cell biocatalysis with recombinant *E. coli* JM109. *Solid line* separation of 2 mM *N*Carb*p*NO_2_ *β*Phe standard (*(R/S)*-5a), *dashed line* separation of *N*Carb*p*NO_2_ *β*Phe (*(R/S)*-5a) after 24 h biotransformation. **b** Concentrations of the *(S)*- and *(R)*-enantiomer (*empty circles* and *filled circles*) and enantiomeric excess (*filled triangles*) during the reaction progress. Reactions and measurements were carried out in triplets and error bars show the standard deviations of the means. Detection by HPLC Agilent 1200 system [Chiralpak QN-AX column, 257 nm, 30 °C, 0.3 mL/min; isocratic mobile phase: 98 % (v/v) methanol (0.2 % v/v ammonium formate) and 2 % (v/v) acetic acid (0.2 M, adjusted to pH 6 with ammonia)]

As shown above, by performing whole cell biotransformation reactions, the hydrolysis of *p*NO_2_PheDU (**4a**) via *A. crystallopoietes* hydantoinase was proven. To investigate the enantiopreference of this enzyme toward the novel substrate, HPLC analytics with chiral stationary phases have been established. The enantiomers of the product were successfully separated with retention times of 13.2 and 14.4 min for the *(S)*- and *(R)*-enantiomer (see Fig. 3a).

By comparing the separation of the *N*Carb*p*NO_2_*β*Phe (*(R/S)*-**5a**) standard with the product formed after 24 h of biotransformation, a preference of the hydantoinase for the *(S)*-enantiomer was observed, consistent to the results reported before. More precisely, 0.77 mM of *(S)*-*N*Carb*p*NO_2_*β*Phe (*(S)*-**5a**) emerged after 24 h (see Fig. 3b). However, only 0.08 mM of the *(R)*-enantiomer were detected after a reaction time of 24 h. Consequently, within the first 5 h of whole cell biotransformation an enantioselectivity of >99 % ee was achieved, while after 24 h the enantiomeric excess was 80.4 % ee. Control experiments without cells do not reveal any product formation (data not shown), confirming the conversion of the novel substrate to the corresponding *N*-carbamoyl-*β*-amino acid by the hydantoinase of *A. crystallopoietes* with preference to the *(S)*-enantiomer.

## Discussion

Since the use of enantiopure *β*-amino acids is of increasing interest for applications in pharmaceutical industries, we focused on a modified hydantoinase process, which is based on the well established classical hydantoinase process for the production of enantiopure *α*-amino acids. The viability of the first step in this proposed modified hydantoinase process, the hydrolysis of dihydrouracil as well as the hydrolysis of differently substituted dihydrouracils was shown in previous works (May et al. 1998; Servi et al. 2005; O’Neill et al. 2011).

In this study, the chemical synthesis of the novel functionalized dihydrouracil *p*NO_2_PheDU (**4a**) was accomplished. Additionally, the corresponding *N*-carbamoylamino acid was chemically prepared for analytical issues. Thereupon, the hydrolysis of this substrate by the hydantoinase from *A. crystallopoietes* DSM20117 was investigated in terms of the synthesis of enantiopure *β*-amino acids. Performing whole cell biotransformation experiments, the enzymatic hydrolysis of *p*NO_2_PheDU (**4a**) could be shown with the highest level of specific activity after 2 h with 0.326 mU/mg_cdw_ and a total conversion yield of 72.27 % after 24 h. To exclude a possible chemical degradation or thermal instability of the substrate, control experiments without cells have been conducted. The latter showed no decrease of substrate concentration and therefore the enzymatic hydrolysis of *p*NO_2_PheDU (**4a**) by the hydantoinase from *A. crystallopoietes* DSM20117 was verified. A limiting factor in this approach is the low solubility of the substrate. By addition of 5 % DMSO, we achieved an initial substrate concentration of 2 mM. It remains to be examined whether higher initial substrate concentrations with higher contents of DMSO would lead to increased enzyme activities or enzyme inactivation (Arcuri et al. 2003).

The successful separation of both enantiomers of the product *N*Carb*p*NO_2_*β*Phe allowed investigation of the enantiopreference of this hydantoinase toward *p*NO_2_PheDU (**4a**). During the first 5 h of biotransformation an enantiomeric excess of >99 % ee for the *(S)*-enantiomer was demonstrated, decreasing to 80.4 % ee after 24 h. As mentioned earlier, due to the low solubility of the substrate *p*NO_2_PheDU (**4a**), a maximum final concentration of 2 mM was achieved. Thus, since no substrate saturation was accomplished, we suggest that after hydrolysis of the preferred *(S)*-*p*NO_2_PheDU (**4a**), the hydantoinase starts converting the *(R)*-enantiomer of the racemic substrate. Another alternative is a spontaneous racemization of the product. This has also been monitored by HPLC analytics with chiral stationary phases and no racemization was observed for *(S)*-*N*Carb*p*NO_2_*β*Phe (*(S)*-**5a**) in Tris–HCl (50 mM, pH 8) at 40 °C during 48 h. Consequently, in contrast to the hydantoins tested to date, we could show that the novel substrate *p*NO_2_PheDU (**4a**) has been hydrolyzed with preference of the *(S)*-enantiomer, which is consistent with previous studies concerning other dihydrouracils (O’Neill et al. 2011; Engel et al. 2012b).

However, there is still a limiting factor for synthesis of the enantiopure *β*-amino acid with 100 % yield: the racemization of the applied substrate. As already mentioned, the racemization of dihydrouracils **4** is challenging compared to hydantoins given that no keto-enol-tautomerism occurs. There are not many investigations concerning this topic, but Argyrou et al. examined the interchange of protons in the carbon 5 (C5) and revealed spontaneous racemization of 5-monosubstituted 5,6-dihydrouracils due to the acidic proton at a carbon next to a carbonyl group. In contrast, for dihydroorotate (6-carboxy-dihydrouracil) no racemization was observed (Argyrou and Washabaugh 1999). This was approved for further 6-monosubstituted dihydrouracil derivatives by Martínez-Gómez et al. (Martínez-Gómez et al. 2012). Due to the chosen synthesis strategy for the novel 6-monosubstituted dihydrouracil, until now we could not achieve the synthesis of enantiopure *p*NO_2_PheDU (**4a**) in terms of analyzing the racemization of this dihydrouracil.

Nevertheless, the findings of this work represent a promising basis to employ the novel substrate for the synthesis of optically pure *β*-amino acids by further conversion of the resulting *N*-carbamoyl-*β*-amino acid. In previous approaches, the hydrolysis of *N*-carbamoyl-*β*-amino acids was performed chemically (O’Neill et al. 2011). Therefore it is worthwhile to detect an appropriate carbamoylase (EC 3.5.1.77; EC 3.5.1.87) realizing this reaction to gain *β*-amino acids. *β*-Ureidopropionases (EC 3.5.1.6) may also be prospect enzymes since they catalyze the last step of the reductive pyrimidine degradation pathway, hydrolyzing *N*-carbamoyl-*β*-alanine and *β*-ureidoisobutyric acid to *β*-alanine and *β*-aminoisobutyric acid (Martínez-Gómez et al. 2012).

## Additional file

10.1186/s13568-015-0174-8 NMR-spectra of *p*NO_2_PheDU (**4a**) and *N*Carb*p*NO_2_ *β*Phe (**5a**).
