# Supplementary material for: Chemical synthesis and enzymatic, stereoselective hydrolysis of a functionalized dihydropyrimidine for the synthesis of β-amino acids
Source: AMB Express. 2015 Dec 24;5:85. doi: 10.1186/s13568-015-0174-8 (PMC4690820; doi:10.1186/s13568-015-0174-8)
Supplement: Supplementary file 1 — 10.1186/s13568-015-0174-8 NMR-spectra of pNO2PheDU (4a) and NCarbpNO2 βPhe (5a). [file 13568_2015_174_MOESM1_ESM.pdf]

Supplementary Material

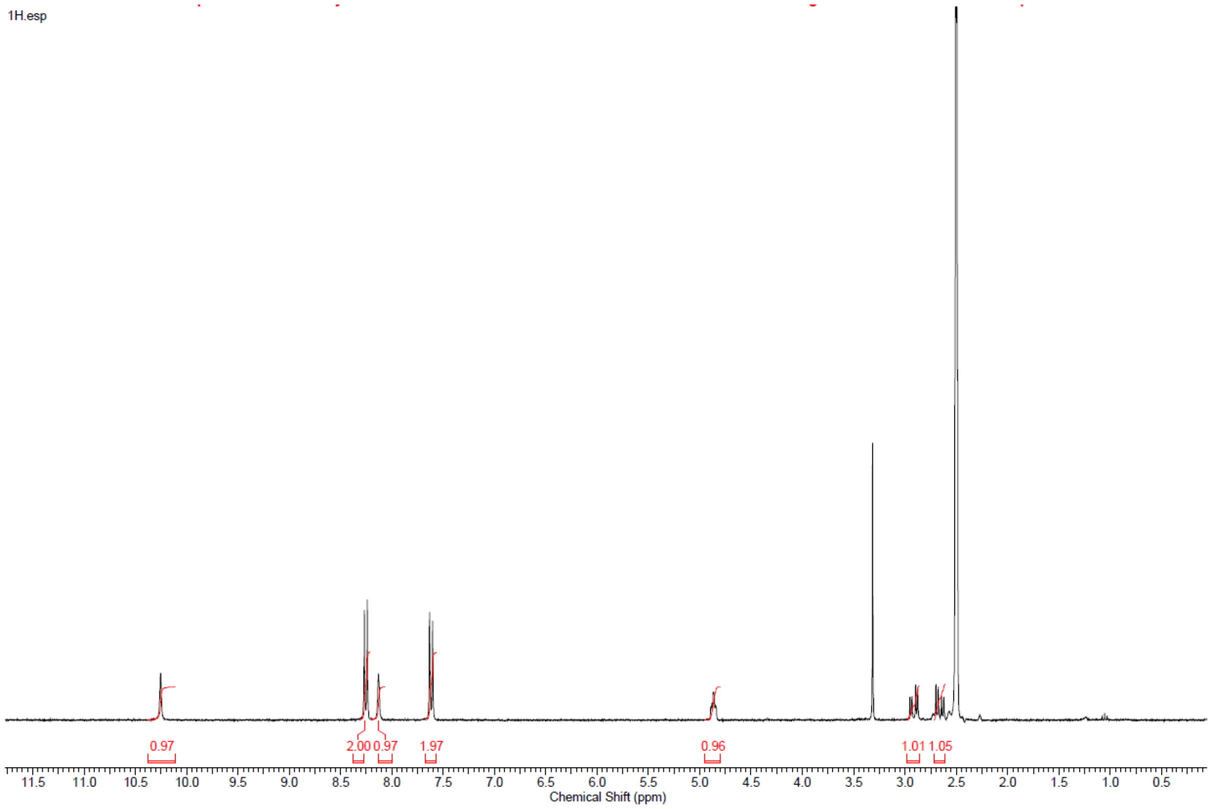

Figure S1 **^{1}H NMR spectrum of compound** 4a**, 400 MHz, DMSO-d$_6$**

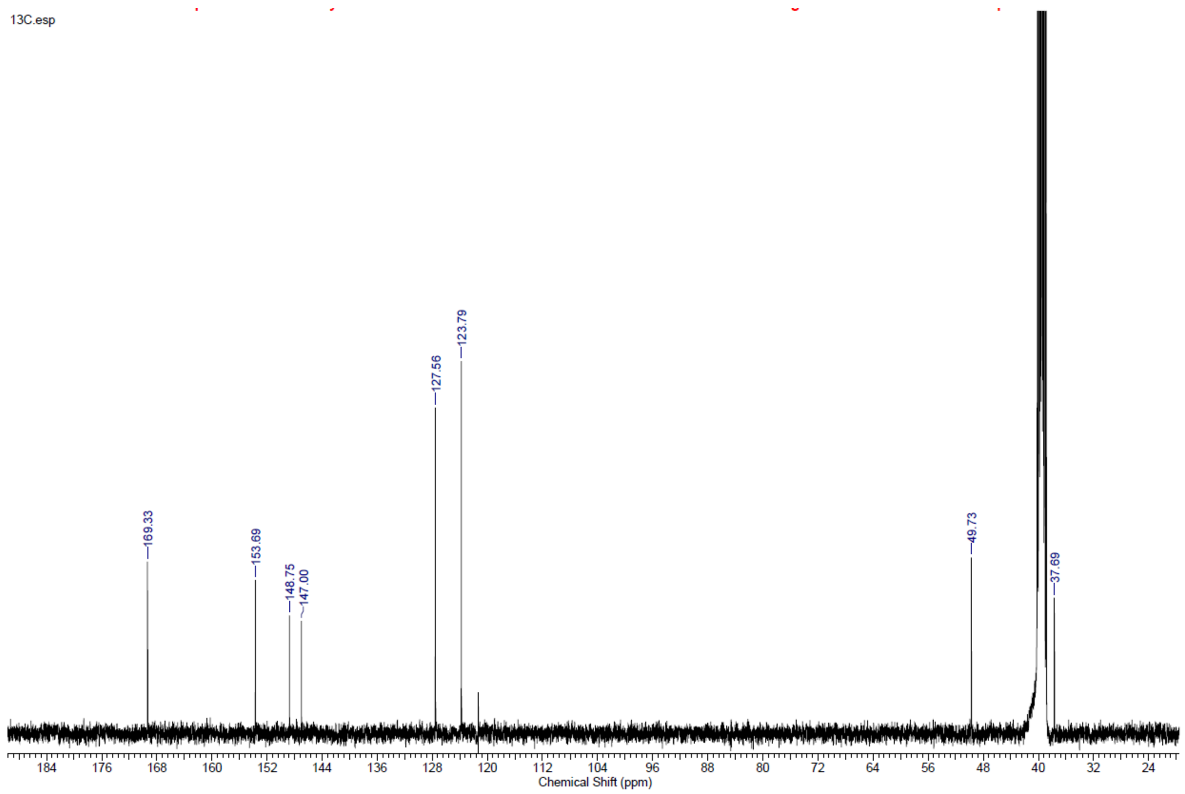

Figure S2 ^{13}C NMR spectrum of compound **4a**, 100 MHz, DMSO-d_6

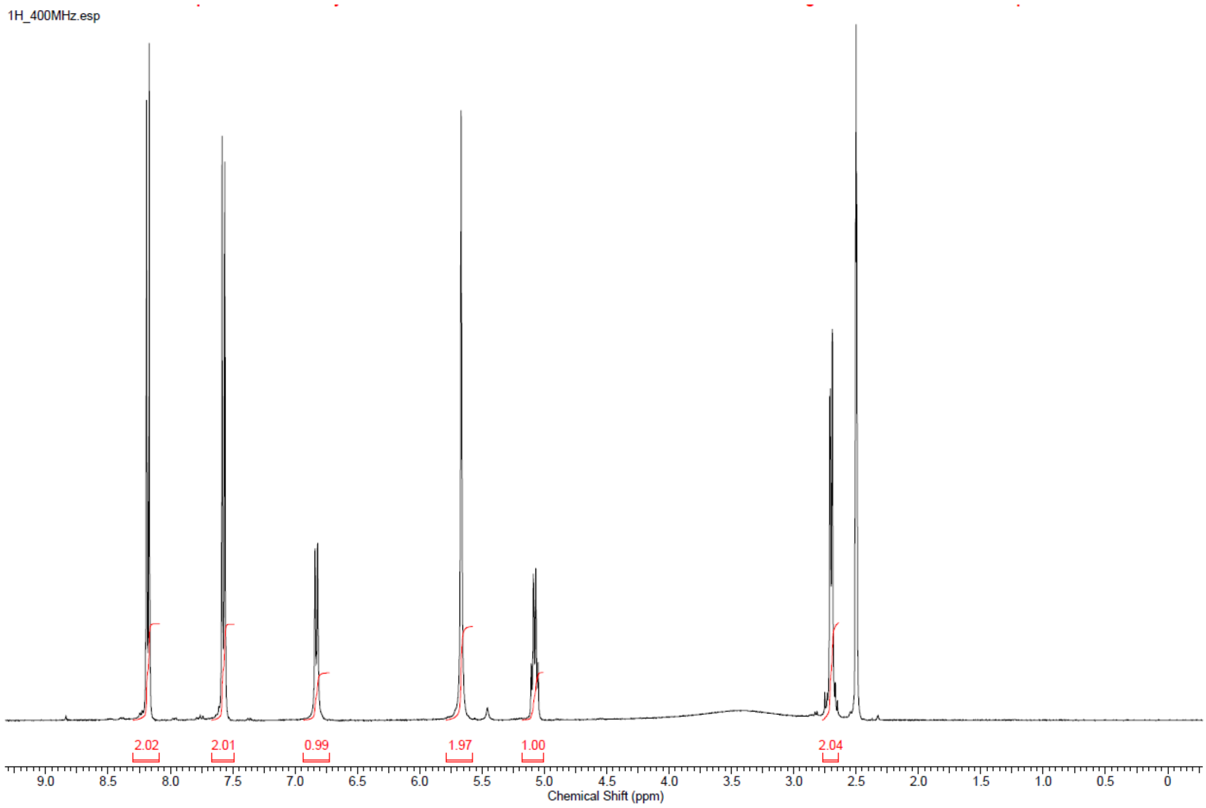

Figure S3 ^{1}H NMR spectrum of compound **5a**, 400 MHz, DMSO-d_6

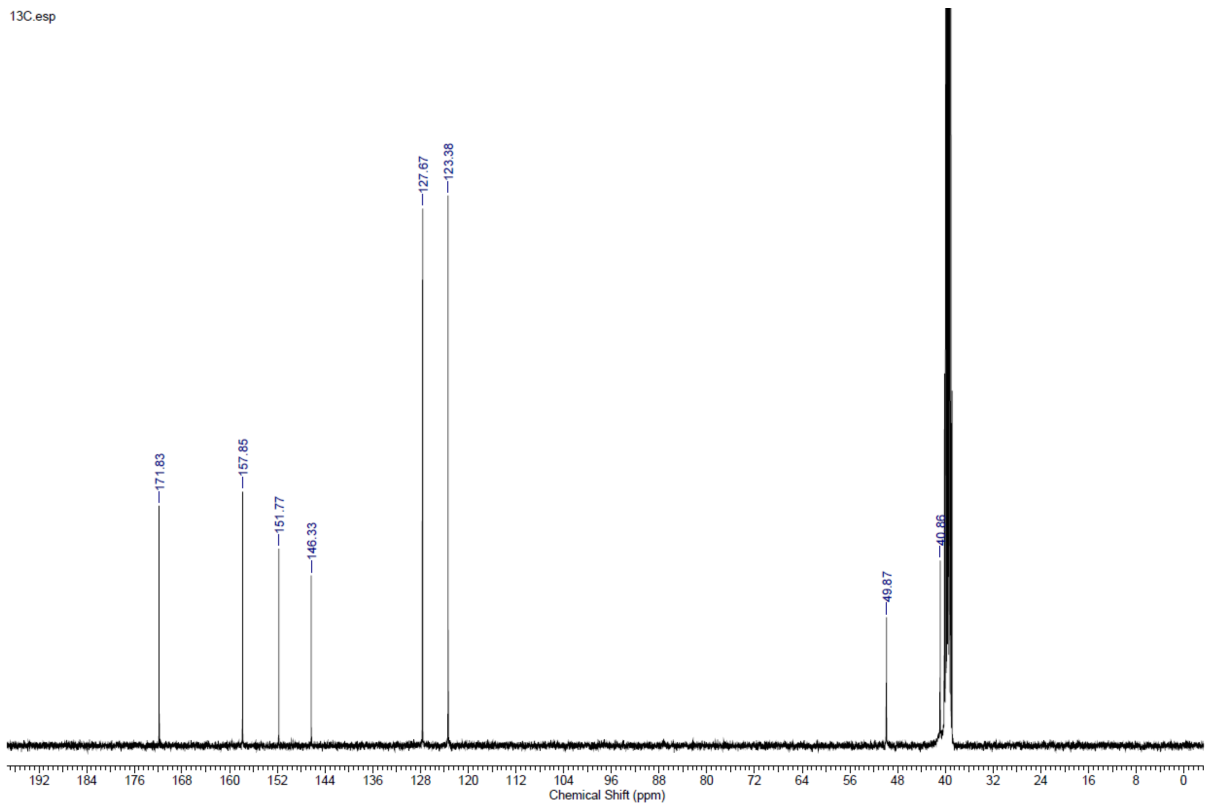

Figure S4 ^{13}C NMR spectrum of compound **5a**, 100 MHz, DMSO-d_6
